# Using fluorescent dissolved organic matter to trace and distinguish the origin of Arctic surface waters

**DOI:** 10.1038/srep33978

**Published:** 2016-09-26

**Authors:** Rafael Gonçalves-Araujo, Mats A. Granskog, Astrid Bracher, Kumiko Azetsu-Scott, Paul A. Dodd, Colin A. Stedmon

**Affiliations:** 1 Alfred Wegener Institute Helmholtz Centre for Polar and Marine Research (AWI), Climate Sciences Division, Physical Oceanography of Polar Seas, Bussestraße 24, 27570 Bremerhaven, Germany; 2 University of Bremen, Faculty of Biology and Chemistry (FB2) - PO Box 330440, 28334 Bremen, Germany; 3 Technical University of Denmark, National Institute for Aquatic Resources, Section for Marine Ecology and Oceanography, Kavalergården 6, 2920 Charlottenlund, Denmark; 4 Norwegian Polar Institute, Fram Centre, Postbox 6606 Langnes, 9296 Tromsø, Norway; 5 University of Bremen, Institute of Environmental Physics, PO Box 330440, 28334 Bremen, Germany; 6 Fisheries and Ocean, Canada, Bedford Institute of Oceanography, PO Box 1006, Dartmouth, Nova Scotia, BY2 4A2 Canada

## Abstract

Climate change affects the Arctic with regards to permafrost thaw, sea-ice melt, alterations to the freshwater budget and increased export of terrestrial material to the Arctic Ocean. The Fram and Davis Straits represent the major gateways connecting the Arctic and Atlantic. Oceanographic surveys were performed in the Fram and Davis Straits, and on the east Greenland Shelf (EGS), in late summer 2012/2013. Meteoric (*f*_*mw*_), sea-ice melt, Atlantic and Pacific water fractions were determined and the fluorescence properties of dissolved organic matter (FDOM) were characterized. In Fram Strait and EGS, a robust correlation between visible wavelength fluorescence and *f*_*mw*_ was apparent, suggesting it as a reliable tracer of polar waters. However, a pattern was observed which linked the organic matter characteristics to the origin of polar waters. At depth in Davis Strait, visible wavelength FDOM was correlated to apparent oxygen utilization (AOU) and traced deep-water DOM turnover. In surface waters FDOM characteristics could distinguish between surface waters from eastern (Atlantic + modified polar waters) and western (Canada-basin polar waters) Arctic sectors. The findings highlight the potential of designing *in situ* multi-channel DOM fluorometers to trace the freshwater origins and decipher water mass mixing dynamics in the region without laborious samples analyses.

Arctic rivers supply high loads of freshwater and dissolved organic matter (DOM) to the Arctic Ocean^1^,^2^,^3^. A major fraction of this DOM, which is mobilized from high latitude carbon-rich soils and peatlands^4^,^5^, is transported across shelf seas^6^ and is widely distributed across the surface waters of the Arctic Ocean. This makes the Arctic Ocean globally unique being highly impacted by both freshwater and terrestrial organic carbon compared to other ocean basins^5^. With the expected permafrost thaw due to the effects of global warming over the Arctic^7^, changes in freshwater export, production of DOM in river catchments and riverine transport of organic material into the shelf seas are foreseen^8^,^9^.

The strong relationship between riverine DOM and freshwater in the Arctic Ocean presents the opportunity of using DOM measurements to isolate and trace the contribution of Arctic riverine freshwater to the Arctic surface waters^10^. Inflow from the Pacific Ocean through the Bering Strait is also an important component of the Arctic Ocean freshwater budget due to its lower salinity^11^,^12^. In addition to regional input from North American and East Siberian rivers the high productivity of the Chukchi shelf results in these waters also having a high DOM signal although less terrestrial in nature^1^. Initial studies have indicated that the optical properties of DOM in surface and halocline (polar) waters of the Eurasian and Canada basin differ^1^ and suggest that there may be potential to utilize this to trace the contribution of these two freshwater sources to water exiting the Arctic Ocean into the North Atlantic through the two major gateways; Fram Strait and the Canadian Arctic Archipelago (CAA)/Davis Strait. It is important to understand the fate and any changes in the export of Arctic freshwater as two major sites of meridional overturning circulation bottom water formation lie directly in recipient waters; the Nordic Seas and the Labrador Sea^13^.

The Fram Strait is characterized by two main currents: to the west, the Arctic outflow carrying the cold polar waters, and to the east the Atlantic inflow^12^,^14^. Additionally, it has been demonstrated that there is recirculation of Atlantic water within the region^15^. During summer, the polar waters are characterized by a shallow surface layer influenced by high fractions of seasonal sea-ice melt forming a low salinity surface layer over the underlying polar waters with brine excess and high fractions of meteoric water (a combination of river water, precipitation and glacial melt)^16^,^17^. After passing through the Fram Strait, the polar waters are transported along the east Greenland shelf by the East Greenland Current (EGC). On the eastern side of Fram Strait, the Atlantic inflow is primarily characterized by warm and saline Atlantic water with little or no influence from meteoric waters^12^,^14^,^17^.

The Davis Strait, at approximately 67°N between Canada and Greenland, represents a transition from Arctic to North Atlantic environments. In the western Davis Strait, the Baffin Island Current (BIC) transports polar waters southwards, towards the Labrador Sea^18^,^19^. These waters have similar characteristics to their equivalent in Fram Strait: relatively low salinity, near freezing temperatures, high meteoric water fractions and brine excess^20^,^21^. The surface waters of eastern Davis Strait are mainly characterized by the presence of the West Greenland Shelf Water (WGSW) and the West Greenland Irminger Water (WGIW). The WGSW originates from the EGC after it turns northward at the southern tip of Greenland, and continues as the West Greenland Current (WGC)^18^,^19^. The WGIW is of Atlantic origin (high temperature and salinity) and is transported northward along the western Greenland slope by the West Greenland Slope Current (WGSC), parallel to WGC^18^,^19^.

A fraction of DOM is colored (CDOM) absorbing light (especially in the ultraviolet – UV – range), and in the Arctic this influences light and heat penetration in surface waters^22^,^23^. When present in high concentrations CDOM imparts a brown color to water easily visible by eye or in satellite ocean color measurements near the mouths of Arctic rivers^6^. Despite considerable dilution the color signal from Arctic riverine CDOM can be easily traced across the Arctic. In addition the spectral properties of the absorption spectrum can be used to differentiate between contrasting CDOM sources such as marine productivity and rivers^1^. A fraction of CDOM also emits a fluorescence signal (hereafter FDOM) which provides not only quantitative information on DOM, but also qualitative information regarding the composition and origin^24^. Fluorescence measurements are well suited for *in situ* sensors and studies have shown that visible wavelength DOM fluorescence (VIS-FDOM) can be linked to Arctic upper halocline waters^3^,^25^ and used to map DOM distribution at higher resolution. Detailed measurement and characterization of DOM fluorescence properties offers the potential to optimize the design and use of these *in situ* fluorometers, which typically measure at single excitation and emission wavelength pairs. Laboratory-based spectroscopic analysis of DOM results in an excitation-emission-matrix (EEM), which maps the UV-visible fluorescence properties. These EEMs represent a combined quantitative and qualitative measure of different signals present in FDOM, which can subsequently be separated into independent underlying DOM components using Parallel Factor Analysis (PARAFAC). Some of those components have been shown to match with fluorescence of specific organic compounds^26^ and are related to some DOM molecular species^27^,^28^. PARAFAC characterization of FDOM has been recently used to assess DOM variability in the Arctic Ocean^29^,^30^,^31^, and here we seek to build on this and link the distribution of different FDOM components to bulk water fractions and mixing. Although having a less sensitive signal in comparison to FDOM^24^,^32^, CDOM has shown to be a robust proxy for halocline (polar) waters^10^,^33^. Based on that this study aims primarily to assess the potential of FDOM, especially VIS-FDOM, as a tracer of polar waters along two important export pathways of Arctic waters: Fram Strait (as well as the eastern Greenland shelf) and Davis Strait. Secondly the biogeochemical dynamics of FDOM was evaluated in Davis Strait. The results here can be further applied on the development of *in situ* profilers, as well as autonomous platforms (such as ROVs and AUVs), focusing on monitoring the freshwater fluxes exiting the Arctic Ocean. Moreover, it would increase the sampling resolution and accelerate the data processing, given that waters samples (especially *δ*^18^O, alkalinity and nutrient analysis) would be taken only for calibration purposes and lab work time would be reduced.

## Results

### Water mass distribution

**Figure 1:**
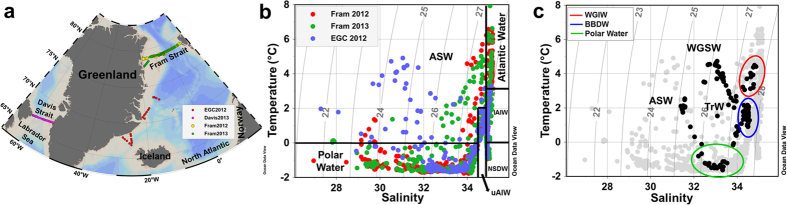
Study area and water masses. (**a**) Map of the study area and sampling stations in 2012 and 2013. (**b**) T-S diagram for all the oceanographic stations (except Davis 2013) considered for this study with the identified water masses^22^,^34^ (Table S1): Atlantic Water, Polar Water, Arctic Surface Water (ASW), upper Arctic Intermediate Water (uAIW), lower Arctic Intermediate Water (lAIW) and Norwegian Sea Deep Water (NSDW). (**c**) T-S diagram showing the eastern Greenland cruises (gray) and Davis 2013 (black) with the identified water masses for the latter region (adapted from *Tang et al.*^18^, *Azetsu-Scott et al.*^21^, *Curry et al.*^19^): West Greenland Shelf Water (WGSW), West Greenland Irminger Water (WGIW), Polar Water, Arctic Surface Water (ASW), Baffin Bay Deep Water (BBDW) and Transitional Water (TrW). Isopycnals [potential density (σ, kg m^−3^)] are indicated as gray lines in (**b**) and (**c**). Produced with Ocean Data View^60^.

**Figure 2:**
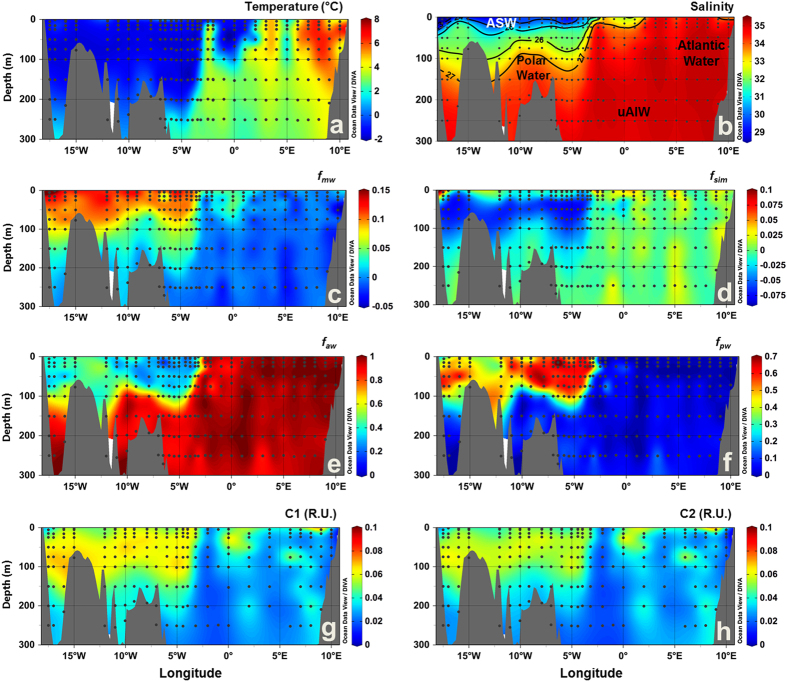
Vertical sections across the surface layer of Fram Strait in September 2012. (**a**) temperature (°C), (**b**) salinity, fractions of (**c**) meteoric water (*f*_*mw*_), (**d**) sea-ice melt (*f*_*sim*_), (**e**) Atlantic water (*f*_*aw*_), and (**f**) Pacific water (*f*_*pw*_), (**g**) C1 (R.U.) and (**h**) C2 (R.U.). In (**b**) black lines indicate the potential density (σ, kg m^−3^) and the abbreviations indicate the position of the water masses defined based on T-S diagrams (Fig. 1). Produced with Ocean Data View^60^.

**Figure 3:**
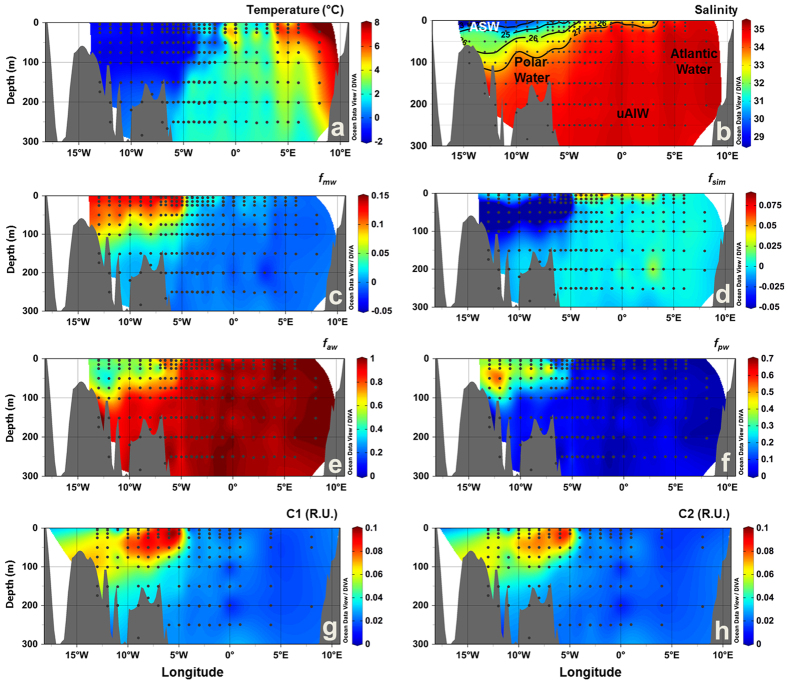
Vertical sections across the surface layer of Fram Strait in September 2013. (**a**) temperature (°C), (**b**) salinity, fractions of (**c**) meteoric water (*f*_*mw*_), (**d**) sea-ice melt (*f*_*sim*_), (**e**) Atlantic water (*f*_*aw*_) and (**f**) Pacific water (*f*_*pw*_), (**g**) C1 (R.U.) and (**h**) C2 (R.U.). In (**b**) black lines indicate the potential density (σ, kg m^−3^) and the abbreviations indicate the position of the water masses defined based on T-S diagrams (Fig. 1). Produced with Ocean Data View^60^.

**Figure 4:**
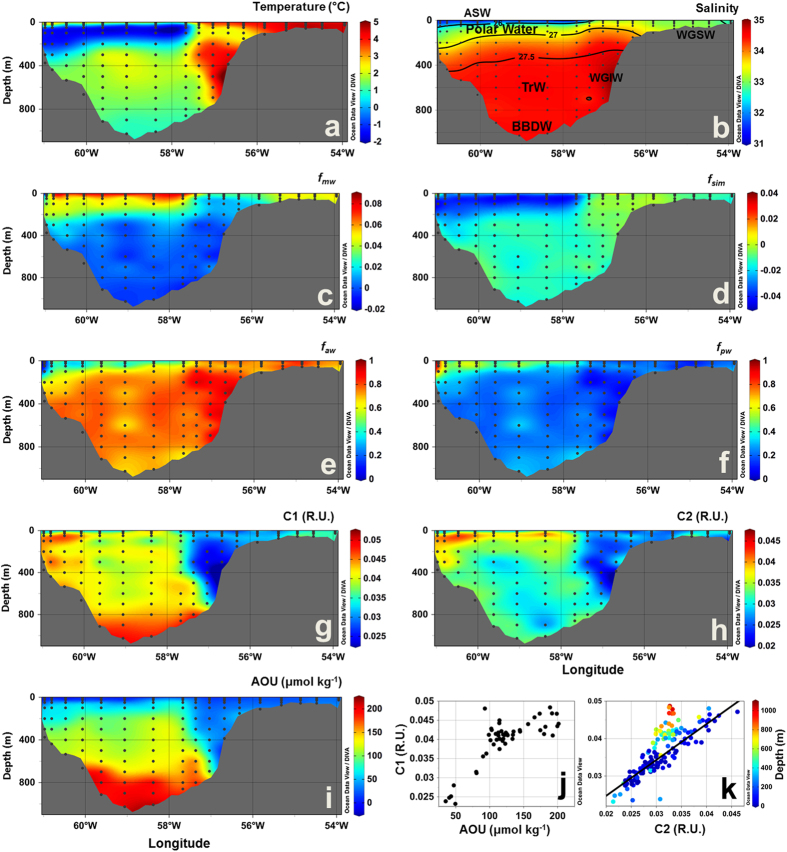
Vertical sections across Davis Strait in September 2013. (**a**) temperature (°C), (**b**) salinity, fractions of (**c**) meteoric water (*f*_*mw*_), (**d**) sea-ice melt (*f*_*sim*_), (**e**) Atlantic water (*f*_*aw*_) and (**f**) Pacific water (*f*_*pw*_), (**g**) C1 (R.U.), (**h**) C2 (R.U.) and (**i**) apparent oxygen utilization (AOU, μmol kg^−1^. (**j**) AOU (μmol kg^−1^) vs. C1 (R.U.) for samples under influence of TrW and BBDW (below 300 m). (**k**) C2 vs. C1 plots for all the samples collected in the Davis Strait 2013, with colorbar indicating depth (**m**). In (**b**) black lines indicate the potential density (σ, kg m^−3^) and the abbreviations indicate the position of the water masses defined based on T-S diagrams (Fig. 1). Produced with Ocean Data View^60^.

Six water masses were identified in Fram Strait, on east Greenland shelf and Iceland Sea, based on published thermohaline characteristics^22^,^34^ (Table S1), as shown on the T-S diagram (Fig. 1b): Atlantic Water, Polar Water and Arctic Surface Water (ASW) in the surface layer (<~200 m); and upper and lower Arctic Intermediate Water (uAIW and lAIW, respectively) and Norwegian Sea Deep Water (NSDW) in the deep layers. In Davis Strait a similar pattern for the temperature versus salinity relation was observed, however with lower salinity values (Fig. 1c). For Davis Strait the following waters masses were observed: West Greenland Shelf Water (WGSW), West Greenland Irminger Water (WGIW), Polar Water, Arctic Surface Water (ASW), Transitional Water (TrW) at depth >300 m and Baffin Bay Deep Water (BBDW) at depth >900 m (adapted from *Tang et al.*^18^, *Azetsu-Scott et al.*^21^, *Curry et al.*^19^). In cruises east of Greenland temperature ranged from −1.77 °C to 7.92 °C with the highest values associated with Atlantic Water in eastern Fram Strait (Figs 1b, 2a and 3a). In Davis Strait the highest temperatures (>3 °C) were associated with WGSW and WGIW (in eastern Davis Strait) whereas the lowest values (down to −1.63 °C) were found within the Polar Water in the western Davis Strait (Figs 1c and 4a). Salinity in Fram Strait and east Greenland shelf varied typically between 28 and 35 with highest salinity associated with Atlantic Water and the deeper waters (>~500 m; lAIW and NSDW), while the lowest values were observed in surface waters in central Fram Strait and inner Greenland shelf (Figs 1b, 2b and 3b). In Davis Strait, salinity ranged from 31.40 to 34.87, with highest salinity in warm subsurface waters of WGIW and TrW (Figs 1c and 4b). BBDW occupied the deepest parts of the Davis Strait section (>750 m) and had lower temperatures than the layer above it, characterized by TrW. The distribution of apparent oxygen utilization (AOU) in Davis Strait showed a clear pattern with lowest values (<60 μmol kg^−1^) in western Greenland and surface waters, whereas these values increase toward the bottom layer reaching up to 216 μmol kg^−1^ within BBDW (Fig. 4i). Although we have sampled for temperature and salinity over the entire water column, in Fram Strait we hereafter focus our results on the surface layer (300 m).

### Dissolved organic matter fluorescence characterization

**Figure 5:**
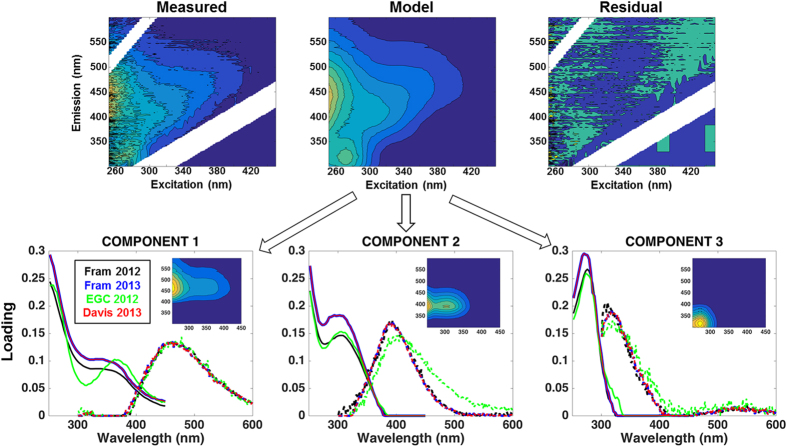
PARAFAC model and isolated components. (**top**) Three-dimensional fluorescence landscapes example of the measured, modeled and residual EEMs of the PARAFAC analysis. (**bottom**) The excitation (solid line) and emission (dashed line) spectra for the three fluorescent components identified by PARAFAC model for each of the cruises. Inset plots show the three-dimensional fluorescence landscapes for each of the final PARAFAC-derived component used in this work (with all cruises merged into one dataset).

Three fluorescent components (C1–C3) were identified during the different PARAFAC runs. C1 and C2 had broad emission and excitation spectra, with emission maxima at visible wavelengths, whereas C3 had an emission maximum at ultraviolet-A wavelengths (UV-A) (Fig. 5, bottom panel). The fluorescence intensities of C1 and C2 ranged from 0 to 0.1 and to 0.09 R.U., respectively, with highest values observed in the polar waters in Fram Strait (Figs 2g,h and 3g,h). In Davis Strait, C1 and C2 fluorescence values were notably lower, only reaching 0.05 and 0.04 R.U., respectively (Fig. 4g,h). In surface waters (depth <300 m) C1 and C2 were significantly correlated (C1 = 1.109 * C2 + 0.001; r^2^ = 0.99; p < 0.0001), however, this correlation was not apparent in Davis Strait deep waters (Fig. 4k). There was a clear addition of C1 in TrW and BBDW, without a proportional increase in C2.

The UV-A fluorescence signal of C3 ranged typically from 0 to 0.04 R.U. and was independent of C1 or C2. Its fluorescence was linked to productivity in surface waters, rather than water mass distribution, as evident from the significant correlation between C3 and chlorophyll-a fluorescence (r^2^ = 0.65, p < 0.0001; Figure S2c). Across the region fluorescence intensities of C3 were generally higher in surface waters (Figure S2b) and profiles often exhibited maxima at or just below phytoplankton chlorophyll fluorescence maxima (Figure S2a).

### Distribution of water fractions

In Fram Strait and on east Greenland shelf *f*_*mw*_ and *f*_*pw*_ followed the distribution patterns of C1. The highest values for *f*_*mw*_ and *f*_*pw*_ were observed on the Greenland shelf, associated with the cold, high DOM, polar waters exiting the Arctic (Figs 2c,f and 3c, 3f). These waters also had negative *f*_*sim*_ values indicating the fact that freshwater has been lost to sea-ice formation and they have experienced brine accumulation in the Arctic Basin (Figs 2d and 3d). In surface waters *f*_*sim*_ was generally less negative or even positive representing the contribution of freshwater from seasonal sea-ice melt. Warmer waters off the Greenland shelf and further east were largely of Atlantic origin with high *f*_*aw*_ (Figs 2e and 3e). Pacific water contribution (*f*_*pw*_) to the polar waters on the Greenland Shelf in Fram Strait was significantly higher in 2012 than in 2013 (p < 0.001) (Figs 2f and 3f).

Some similarities in the distribution of the waters masses in Fram Strait could be observed in Davis Strait (Fig. 4c–f). In western Davis Strait, cold polar waters occupied the sub-surface layer, characterized by sub-zero temperatures and high contribution of *f*_*mw*_ (Fig. 4c). Similarly the highest *f*_*sim*_ values were at the very surface (0–30 m), indicating sea-ice melt, and the lowest (negative values) were associated with the polar waters in western Davis Strait (Fig. 4d). The *f*_*aw*_ was the most dominant fraction on the west Greenland shelf and in deeper waters (Fig. 4e). The contribution of Pacific water (*f*_*pw*_) was associated with the cold polar waters exported from the Arctic (Fig. 4f).

### Linking visible organic matter fluorescence to water fractions

**Figure 6:**
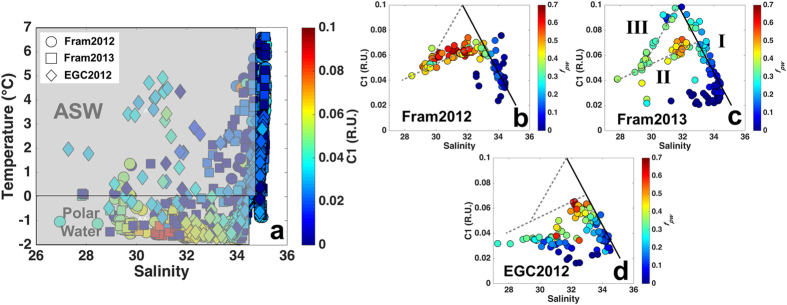
T-S diagram and correlations between salinity and C1 in the east of Greenland. (**a**) Temperature (°C) vs. salinity with colorbar indicating C1 (R.U.) for all the samples collected in the eastern Greenland cruises. (**b–d**) Salinity vs. C1 (R.U.) and *f*_*pw*_ as colorbar for polar waters and ASW for each of the eastern Greenland cruises. Black solid line (I) indicates the mixing curve for the polar waters (based on Fram 2012 and 2013 datasets). Gray dashed lines (II and III) indicate the two distinct mixing curves of polar waters over the Greenland shelf. The regressions were obtained by combining the three datasets. (I) y = –0.02 *(Sal) + 0.723, r^2^ = 0.90, p < 0.0001, n = 240. (II) y = 0.0042 *(Sal) − 0.0698, r^2^ = 0.90, p < 0.0001, n = 126. (III) y = 0.0183 *(Sal) −0.4816, r^2^ = 0.98, p < 0.0001, n = 18.

**Figure 7:**
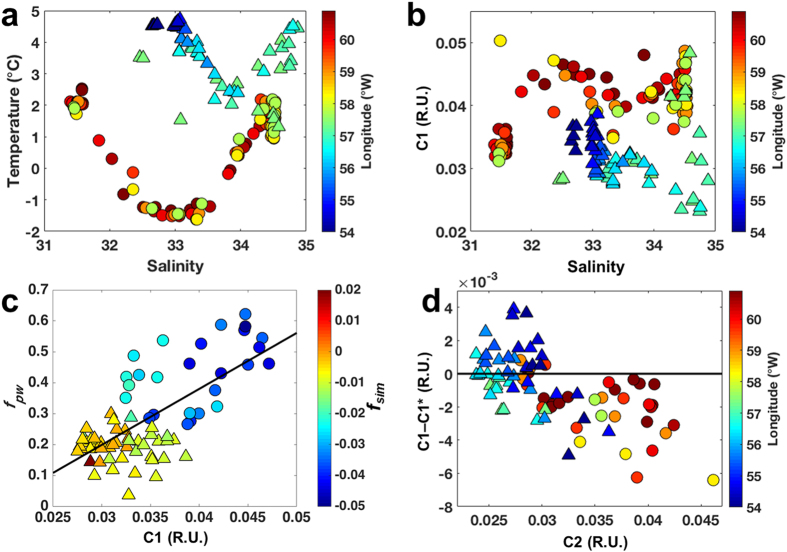
VIS-FDOM as a water mass tracer in the Davis Strait. Plots for the Davis2013 cruise. (**a**) T-S diagram with longitude (°W) as colorbar. (**b**) Salinity vs. C1 (R.U.), with colorbar indicating longitude (°W). (**c**) C1 (R.U.) vs. *f*_*pw*_ for the surface layer (<300 m) and *f*_*sim*_ as colorbar. (**d**) C2 (R.U.) vs. C1–C1* (R.U.) for the surface layer (<300 m), with longitude (°W) as colorbar. Triangles indicate the samples within the eastern part of Davis Strait, whereas circles refer to samples located in the western sector (separated by the 57.5 °W longitude). Black line in (**c**) indicate the best fit.

The T-S diagram (Fig. 6a) shows a clear distinction of polar waters exiting the Arctic, with respect to C1. Highest C1 fluorescence was associated with polar waters and ASW. The latter had comparatively lower values, indicating the dilution of surface waters by sea-ice melt and precipitation (glacial input and snow). The correlation of C1 with both temperature (not shown) and salinity (Fig. 6b–d) presented a very similar, however tighter, pattern than portrayed by absorption alone^10^,^33^. When considering the salinity versus C1 relation for each cruise individually (except for Davis Strait), two distinct mixing curves for the dilution of polar waters are apparent (Fig. 6). C1 was also strongly inversely correlated to *f*_*sim*_ (Figure S3) linking the high DOM signal to brine. In Davis Strait, different patterns were observed. The relationships C1 and C2 vs. salinity indicate two mixing curves (Fig. 7) in agreement with the mixing curves visible on the T-S diagram (Fig. 7a,b), where a clear separation of stations from eastern and western Davis Strait is apparent. The correlation between C1 and C2 in the East Greenland data could be harnessed tested if the FDOM in the Davis Strait had the same characteristics (relative proportions of C1 and C2) and hence similar origins. A regression was derived for C1 fluorescence based on C2 considering all the surface data (<200 m). This was then applied to the Davis Strait data to predict expected C1 fluorescence, C1*, for the surface layer in Davis Strait. The difference between measured and predicted C1 fluorescence, C1–C1*, is plotted against C2 (Fig. 7d) and indicates significant differences (p < 0.05) between eastern and western Davis Strait DOM. Samples in eastern Davis Strait have similar properties to those from the Fram Strait, whereas on the Canadian side of the strait the DOM has comparatively less C1. Finally for Davis Strait deep waters (>300 m), C1 was highly correlated with AOU, with the highest values of both parameters in BBDW (Fig. 4g,i). C2 showed no indication of elevated values at depth (Fig. 4h).

## Discussion

The distribution of the water fractions in the surface layer (0–300 m) of the Fram Strait followed the overall patterns and values reported for the region^17^,^33^,^35^ (Figs 2c–f and 3c–f). Similar hydrographic features were also observed in the distributions of temperature and salinity (Figs 2ab and 3ab), agreeing with previous reports^12^,^14^,^22^. *f*_*mw*_ was related to the Arctic outflow through the EGC and the highest values (up to 0.15) were observed in the western section and aligned with earlier reports^10^,^14^,^17^,^33^. Evidence for sea-ice melt was apparent in the surface layer with generally more positive *f*_*sim*_ values than immediately below. *f*_*sim*_ and *f*_*mw*_ were inversely correlated in polar waters indicating the origins from brine rejection during sea ice formation on coastal waters influenced by riverine inputs^10^,^17^,^36^. The *f*_*pw*_ was associated with polar waters with values up to 0.7, and within the range reported in previous multi-year analysis conducted in the region^17^. Interannual variability in the contributions of *f*_*pw*_ to polar waters exiting the Arctic Ocean in the Fram Strait is related to variability in atmospheric forcing, and consequently ocean surface circulation, over the Arctic^35^,^37^.

The three fluorescent components identified by PARAFAC modeling (Fig. 5) are similar to fluorescent components identified in previous studies conducted in Fram and Davis Straits^38^,^39^,^40^, but also in other regions of the Arctic Ocean^29^,^41^. The visible wavelength fluorescence character of C1 and C2 has been linked to aromatic, high molecular weight organic matter (humic-like) with terrestrial character^27^,^28^ and correlated to lignin phenol concentrations^25^. However, the precise chemical origin of those signals is currently unknown and the subject of much research. In Fram Strait, these components (C1 and C2; Figs 2g,h and 3g,h) presented similar distribution as CDOM (*a*_350_)^10^,^22^,^33^. Their fluorescence intensities were highly correlated and both had their maximum associated with the relatively low salinity polar waters and ASW (Fig. 6a) in agreement with previous *in situ* VIS-FDOM measurements (Ex: 350–460 nm; Em: 550 nm) in the region^25^.

The UV-A FDOM signal (C3) is associated with compounds with lower aromaticity, such as dissolved and combined amino acids^42^ and is often linked to aquatic productivity^39^,^40^,^43^,^44^,^45^. As can therefore be expected C3 fluorescence in this study was not correlated to polar waters; but rather linked to phytoplankton productivity in surface waters (Figure S2). In support of this C3 fluorescence in Greenland shelf waters are correlated to amino acid concentrations [Jørgensen & Stedmon, *unpublished data*].

In Davis Strait the distributions of temperature and salinity followed previous reports^18^,^19^,^21^,^46^ (Fig. 4a,b). The surface layer in western Davis Strait was occupied by sub-zero temperature polar waters, characterizing the Arctic outflow with the BIC. Similarly to the Fram Strait, the impact of freshening by seasonal sea-ice melt was observed in a shallow surface layer (~40 m)^19^,^21^. The bottom layer was characterized by the presence of BBDW^21^. While the origin of this water mass is still under debate^18^ the high AOU values (over 220 μmol kg^−1^) associated with it (Fig. 4i,j) are comparable to AOU values observed for very old deep ocean waters and waters beneath productive upwelling regions^43^.

The distribution and contribution of water fractions in Davis Strait were in agreement with previous studies applying different approaches^20^,^21^,^46^ (Fig. 4c–f). As in Fram Strait, polar waters were found in the western sector, with the highest values of *f*_*mw*_ and *f*_*pw*_^20^,^21^,^46^. However, *f*_*pw*_ contributions were greater than the ones found in Fram Strait, with values for polar waters varying between 0.5 and 1, indicating a great contribution of polar waters originating from the Canada basin. The lowest values of *f*_*sim*_ were associated with the polar waters, reflecting the fact that they have been modified by sea-ice formation. This layer was underneath a thin surface layer highly influenced by sea-ice melt^20^,^21^,^46^. The contribution of *f*_*aw*_ was highest in the eastern Davis Strait, associated with the WGC^21^.

The distribution of the components C1 and C2 in Davis Strait surface waters resembled the general hydrographic conditions in the region^19^,^21^,^46^ with the highest fluorescence intensities associated with polar outflow to the west, as portrayed in the Fram Strait. Those components were, however, found in lower concentrations than in Fram Strait polar waters. This can be due to either a greater dilution of polar waters from Canada basin passing through the CAA and Baffin Bay^38^ or an indication of lower FDOM levels in the source Canada basin polar waters relative to Eurasian Basin polar waters. The elevated levels of C1 and C2 observed on the west Greenland shelf likely originates from the diluted, reminiscent FDOM signal from polar waters transported through Fram Strait, with the EGC and subsequently the WGC (see discussion later). Although there is a detectable input of meteoric water from eastern Greenland to the EGC, there is little terrestrial DOM contribution from Greenland to shelf waters^10^.

The fluorescence intensities of C1 and C2 were highly correlated in the whole dataset; however, there were two clear exceptions. In Davis Strait deep waters there had an excess C1 relative to C2. Organic matter with these spectral characteristics has previously been linked to bacterial biomass^47^,^48^, microbial respiration and degradation of organic material^45^,^49^. Earlier studies have linked the generation of visible wavelength FDOM to AOU in ocean bottom waters^43^,^49^, which was also proven by incubation experiments^39^. A similar correlation is apparent in the deep layer of the Baffin Bay for C1 vs. AOU (Fig. 4j). Since ~90% of the oxygen consumption in the deep ocean is due to particle remineralization^50^, our results thus suggest that the observed increase in C1 at the bottom layer is likely derived from the turnover of sinking particulate organic matter. This is supported by the fact that waters from the deeper layers of Davis Strait have a relatively long residence time^51^ where such a signature from the microbial production of bio-refractory material would persist and be easily detectable.

**Figure 8:**
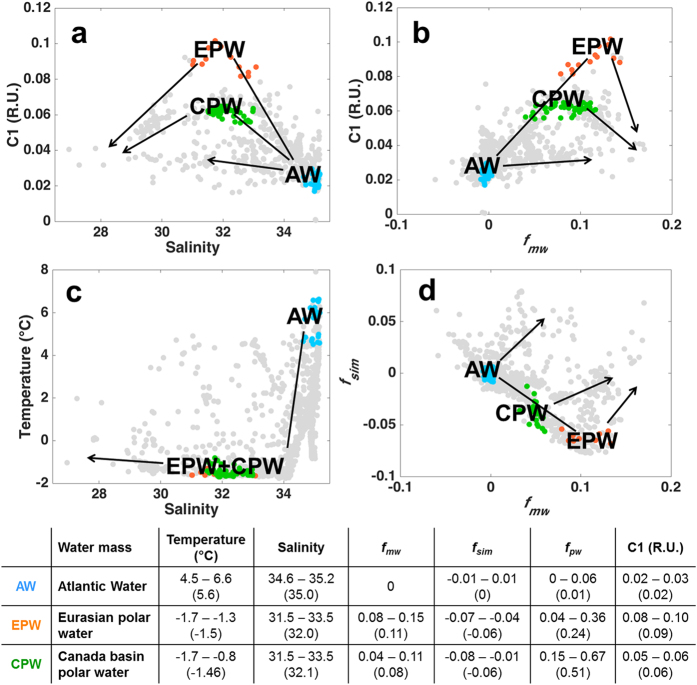
Schematic graphs for eastern Greenland. Schematic graphs showing the behavior during mixing of distinct waters defined in the text (Atlantic water, Eurasian and Canada basin polar waters, whose end members in this study are colored accordingly): for (**a**) C1 and salinity, (**b**) C1 and *f*_*mw*_, (**c**) temperature and salinity, (**d**) *f*_*sim*_ and *f*_*mw*_. All data used in this study is shown with gray dots. Lines indicate the mixing between different waters, whose end-members for this study are tabulated below. Arrows represent the approximate direction of the deviation expected by dilution with sea-ice melt and precipitation (including glacial melt). The table shows information (range and average) on some parameters for the end members of each water type identified in this study.

The second exception to the correlation between C1 and C2 was in the surface waters of the western Davis Strait (Fig. 4k). If the DOM fluorescence signal in polar waters present in Davis Strait and Fram Strait would have common origins one would expect all data to lie on one relationship as dilution would influence both C1 and C2 in the same fashion. The fact that the DOM in the WGC has the same proportions of C1 and C2 as that found in polar waters of the EGC (Fig. 7d) strongly suggests that it represents here the same material transported along the Greenland shelf and gradually diluted. In contrast, the lower levels of C1 relative to C2 in polar waters in the western Davis Strait suggest a different DOM source (Fig. 7d). This could be reflecting the documented differences in DOM in polar waters originating from the Canada and Eurasian Basins, marine production and terrestrial material, respectively^1^. This is supported by the correlation of C1 fluorescence to *f*_*pw*_ in Davis Strait (Fig. 7c) and to *f*_*mw*_ in Fram Strait (Fig. 8b).

In Fram Strait Pacific water contribution varied between 2012 and 2013. Although the Davis Strait results discussed above suggest that visible wavelength DOM fluorescence might distinguish between polar waters from Eurasian and Canada basins, there were no such systematic deviations in Fram Strait C1 vs. C2 relationship, which could be linked to Pacific water contribution. However, plots of C1 fluorescence against salinity and *f*_*mw*_ clearly reveal a segregation into three groups where polar waters highly influenced by *f*_*pw*_ (waters from Canada basin) have lower C1 fluorescence than those of Eurasian origin which have a C1 fluorescence greater than 0.08 R.U. (Figs 6 and 8). Such clear distinction between the origins of polar waters is not apparent for CDOM (*a*_350_)^10^,^33^, most likely due to the lesser sensitivity of this bulk measurement.

Freshening of polar waters at the very surface layer (<40 m) was clearly detected in the relationship between *f*_*mw*_ and *f*_*sim*_ (Fig. 8d), where dilution of both Atlantic and polar waters by sea-ice melt at the surface layer is apparent^10^,^33^,^52^. Dilution of CDOM absorption (*a*_350_) was observed in previous studies where samples deviating from the correlation line (to *f*_*mw*_) indicated the dilution by sea-ice melt and/or precipitation (at the very surface layer)^10^,^33^. However, the correlations observed for fluorescence in this study had a better fit than the ones for *a*_350_. This can again be expected due to the general higher sensitivity of fluorescence measurements in comparison to absorbance spectroscopy^32^. Thus, we surmise VIS-FDOM is a more reliable tracer of polar waters and the mixing processes associated to those waters (sea-ice melt and sea-ice formation). This result holds great promise for further developments in the use of DOM visible wavelength fluorescence in tracer studies in the Arctic and warrants further investigation.

## Summary

The visible wavelength DOM fluorescence components identified by PARAFAC modeling were correlated to the fraction of meteoric and Pacific water determined using established techniques^17^,^53^. The ratio of the two fluorescence signals was linked to the dominant organic matter sources in polar waters exiting the Arctic form the Canada and Eurasian basins. In 2012 a greater fraction of Pacific waters in the Fram Strait suggests greater contribution of waters from the Canada basin which is reflected in organic matter fluorescence intensities. Such changes were not detectable from CDOM absorption measurements^10^,^33^. Our results demonstrate that Eurasian polar waters have higher visible wavelength DOM fluorescence signal than waters from the Canada basin. The result also show that the organic matter exported through the Davis and Fram straits differ in quality reflecting the contrasting dominant sources of DOM in polar waters from the two basins. In addition, in deep waters of the Davis Strait there was a production of bio-refractory organic matter fluorescence signal linked to microbial respiration driven by degradation of sinking particulate matter.

The results presented here provide an indication of which wavelength regions of DOM fluorescence carry information on DOM source and mixing. As fluorescence is well suited for use *in situ* instrumentation, these measurements can aid the design of new multi-channel fluorometers for different platforms. These can provide additional insight into the physical oceanography of the region and complement current hydrographic measurements focused on monitoring freshwater fluxes and circulation.

## Methods

### Sampling strategy

Samples for salinity, dissolved organic matter fluorescence (FDOM), dissolved inorganic nutrients (nitrate and phosphate) and *δ*^18^O were collected during several cruises around Greenland (Fig. 1a). Two cruises were along a section in the Fram Strait at 78°55′N in Aug/Sep of 2012 and 2013 onboard *R*/*V* Lance, hereafter referred to as Fram2012 and Fram2013, respectively. A cruise onboard *R*/*V* Dana (September 2012, hereafter EGC2012) collected samples in the Denmark Strait region, Iceland Sea and along a number of sections across the EGC. Data from Fram2012 and EGC2012 cruises (including hydrography, water fractions and CDOM absorption) are also presented in other study^10^. In addition, samples were collected across the Davis Strait onboard *R*/*V* Knorr (September 2013, hereafter Davis2013). During all cruises temperature and salinity profiles were acquired with a CTD attached to a rosette system at all the stations, which was calibrated with salinity from water samples.

### Analyses of salinity, dissolved inorganic nutrients, dissolved oxygen and *δ* ^18^O

For calibration of the CTD, salinity samples were collected in glass bottles and analyzed using a Guildline 8410A Portasal salinometer (Fram and EGC). For the Fram2012, Fram2013 and EGC2012 cruises, nutrient samples were collected directly into acid-washed polyethylene bottles and frozen immediately after collection, and were kept at −20 °C until analysis. Nutrient analyses were conducted at Aarhus University (Roskilde, Denmark) using an autoanalyzer (Skalar)^54^. For those cruises, *δ*^18^O samples were collected in 40 mL glass vials that were filled completely, closed tightly and sealed with Parafilm, and were analyzed by equilibration with carbon dioxide. Measurements were carried out with isotope ratio mass spectrometers at the G.G. Hatch Stable Isotope Laboratory, University of Ottawa, Canada (Thermo Delta Plus XP).

For the Davis 2013 cruise nutrient samples were frozen and later analyzed at Bedford Institute of Oceanography, Canada, following the World Ocean Circulation Experiment (WOCE) protocols using a Technicon Autoanalyzer with the precision of 0.19 mmol kg^−1^ for nitrate and nitrite (NO_3_ + NO_2_), and 0.04 mmol kg^−1^ for phosphate (PO_4_). Oxygen isotope samples were collected in 60 mL Amber Boston Rounds with Poly-Seal-Lined caps secured with electrical tape, stored at room temperature. They were analyzed with a FISONS PRISM III with a Micromass multiprep automatic equilibration system at Lamont-Doherty Earth Observatory, USA. Two-milliliter subsamples were equilibrated with CO_2_ gas (8 h at 35 °C). Data are reported with respect to standard mean ocean water (SMOW) with the *δ*^18^O notation. The external precision based on replicates and standards is ±0.033‰. Additionally, 293 samples for dissolved oxygen were collected only in the Davis 2013 cruise and analyzed using Winkler titration (with precision of 0.5%), to calibrate oxygen sensors on CTD.

### DOM samples processing

Water samples for DOM analysis (CDOM and FDOM) were collected through prerinsed 0.2 μm Millipore Opticap XL filter capsules, except on the EGC2012 cruise precombusted GF/F filters (nominal pore size 0.7 μm) were used. The samples were stored in pre-combusted amber glass vials in dark at 4 °C until analysis at the Technical University of Denmark, within two months of collection (Fram and Davis Straits) or analyzed immediately onboard (EGC2012). It should be noted that the optimal situation would be to have all samples 0.2 μm filtered (removing bacteria and colloids) and analyzed immediately onboard however, logistical constraints and practicalities of collaborative sampling hindered this. An analysis of histograms of the fluorescence properties of DOM from the Fram Strait (sterile filtered and stored) and the EGC (GFF and analyses immediately) indicated no clear systematic bias resulting from the two approaches.

### Spectroscopic measurements and PARAFAC modeling

CDOM absorbance was measured across the spectral range from 250 to 700 nm using a Shimadzu UV–2401PC spectrophotometer and 100 mm quartz cells with ultrapure water as reference^55^. Absorbance was used to correct fluorescence EEMs.

Fluorescence EEMs were collected using an Aqualog fluorescence spectrometer (HORIBA Jobin Yvon, Germany). Fluorescence intensity was measured across emission wavelengths 300–600 nm (resolution 1.64 nm) at excitation wavelengths from 250 to 450 nm, with 3 nm increments, and an integration time of 8 s. EEMs were corrected for inner-filter effects and for Raman and Rayleigh scattering^56^ (Fig. 5, top panel). The underlying fluorescent components of DOM in the EEMs were isolated by applying PARAFAC modeling using the “drEEM Toolbox”^56^. In this study different PARAFAC model fits were explored. At first, individual PARAFAC models were derived and split-half validated for each cruise individually. The split-half analysis consists in producing identical models from independent subsamples (halves) of the dataset, generally randomly generated. Similar PARAFAC components were identified (Fig. 5, bottom panel) and these results were then compared to a model derived on the combined dataset (1022 samples). The fluorescent components derived from PARAFAC modeling were compared with PARAFAC components from other studies using the OpenFluor database^57^.

### Water masses fractionation

The fractions of meteoric water (*f*_*mw*_), sea-ice melt water (*f*_*sim*_), Pacific seawater (*f*_*pw*_) and Atlantic seawater (*f*_*aw*_) in discrete water samples were derived using a combination of procedures established by *Östlund and Hut*^58^ and *Jones et al.*^11^ as described in *Dodd et al.*^17^. The details behind the choice of end-member values and for the sensitivity of the estimates of freshwater fractions to variations in the end-member composition can be found in *Jones et al.*^16^, *Dodd et al.*^17^ and *Hansen et al.*^59^. In brief, the contribution from Atlantic water, Pacific water, meteoric water, and sea-ice melt was carried out with the following equations:


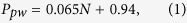



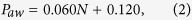



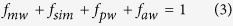



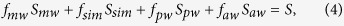



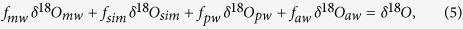



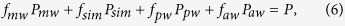


N and P in the equations above correspond to the nitrate and phosphate concentrations, respectively (Figure S1a). The salinity (S) of meteoric water, sea-ice melt, Pacific water, and Atlantic water were 0, 4, 32.0, and 34.9, respectively, and the *δ*^18^O end-members –18.4, 0.5, –1.3, and 0.3, respectively^17^.

## Additional Information

**How to cite this article**: Gonçalves-Araujo, R. *et al*. Using fluorescent dissolved organic matter to trace and distinguish the origin of Arctic surface waters. *Sci. Rep.* **6**, 33978; doi: 10.1038/srep33978 (2016).

## Supplementary Material

Supplementary Information
