# Supplementary material for: Using fluorescent dissolved organic matter to trace and distinguish the origin of Arctic surface waters
Source: Sci Rep. 2016 Sep 26;6:33978. doi: 10.1038/srep33978 (PMC5035922; doi:10.1038/srep33978)

# - SUPPLEMENTARY INFORMATION -

Using fluorescent dissolved organic matter to trace and distinguish the origin of Arctic surface waters

Rafael **Gonçalves-Araujo**[1,2,3]*, Mats A. **Granskog**[4], Astrid **Bracher**[1,5], Kumiko **Azetsu-Scott**[6], Paul A. **Dodd**[4], and Colin A. **Stedmon**[3]**

[1]Alfred Wegener Institute Helmholtz Centre for Polar and Marine Research (AWI), Climate Sciences Division, Physical Oceanography of Polar Seas, Bussestraße 24, 27570 Bremerhaven, Germany

[2]University of Bremen, Faculty of Biology and Chemistry (FB2) - PO Box 330440, 28334 Bremen, Germany

[3]Technical University of Denmark, National Institute for Aquatic Resources, Section for Marine Ecology and Oceanography, Kavalergården 6, 2920 Charlottenlund, Denmark

[4]Norwegian Polar Institute, Fram Centre, Postbox 6606 Langnes, 9296 Tromsø, Norway

[5]University of Bremen, Institute of Environmental Physics, PO Box 330440, 28334 Bremen, Germany

[6]Fisheries and Ocean, Canada, Bedford Institute of Oceanography, PO Box 1006, Dartmouth, Nova Scotia, BY2 4A2 Canada

*__Corresponding author__: rafael.goncalves.araujo@awi.de - rafaelgoncalvesaraujo@gmail.com
**__Corresponding author:__ cost@aqua.dtu.dk

Table S1. Water masses classification. Thermohaline ranges used to characterize the water masses in the Fram Strait and east Greenland [22,34].

Water Mass	Temperature	Salinity
**Atlantic Water (AW)**	> 3 °C	> 34.9
**Arctic Surface Water (ASW)**	> 0 °C<br>> 2 °C	< 34.4<br>< 34.9
**Polar Water (PW)**	< 0 °C	< 34.4
**Upper Arctic Intermediate Water (uAIW)**	< 2 °C	34.4–34.9
**Lower Arctic Intermediate Water (lAIW)**	0–3 °C	> 34.9
**Norwegian Sea Deep Water (NSDW)**	< 0 °C	> 34.9

Figure S1. Water fractionation. **(a)** Phosphate (μM) vs. nitrate (μM) with the equations and source lines for the Atlantic and Pacific waters [17]. **(b)** Salinity vs. $\delta^{18}O$ (‰) with the end members for Atlantic Water (AW), Meteoric Water (MW) and Sea-ice melt (SIM) and corresponding conservative mixing lines [17].

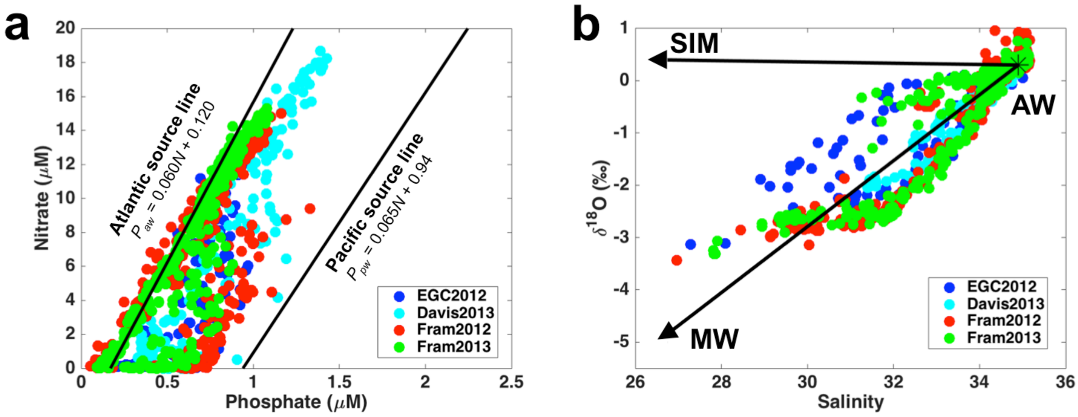

Figure S2. Vertical distribution of Chlorophyll-a and UV-FDOM for the EGC2012 cruise. Vertical distribution of (**a**) chlorophyll-a fluorescence (A.U.) and (**b**) C3 (R.U.) for the EGC2012 cruise, and (c) the correlation between chlorophyll-a fluorescence (A.U.) and C3 (R.U.).

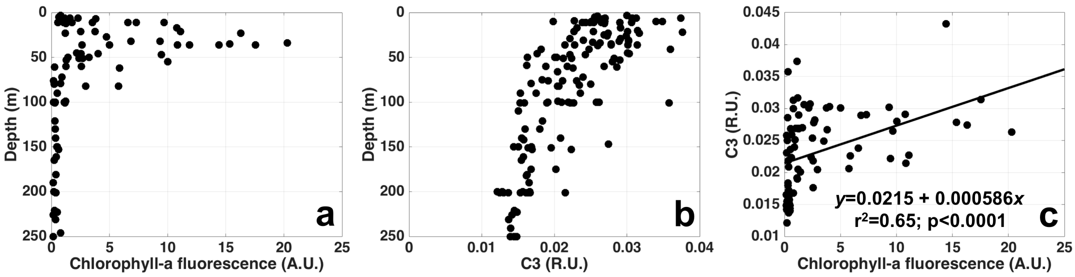

Figure S3. Scatter plots each of the cruises performed in the eastern Greenland, considering only PW and ASW (salinity<34.3). (**top panel**) C1 (R.U.) vs. $\delta^{18}O$ (‰) vs. f_{sim}. (**middle panel**) C1 (R.U.) vs. f_{mw} vs. f_{sim}. (**bottom panel**) C1 (R.U.) vs. f_{sim} vs. salinity.

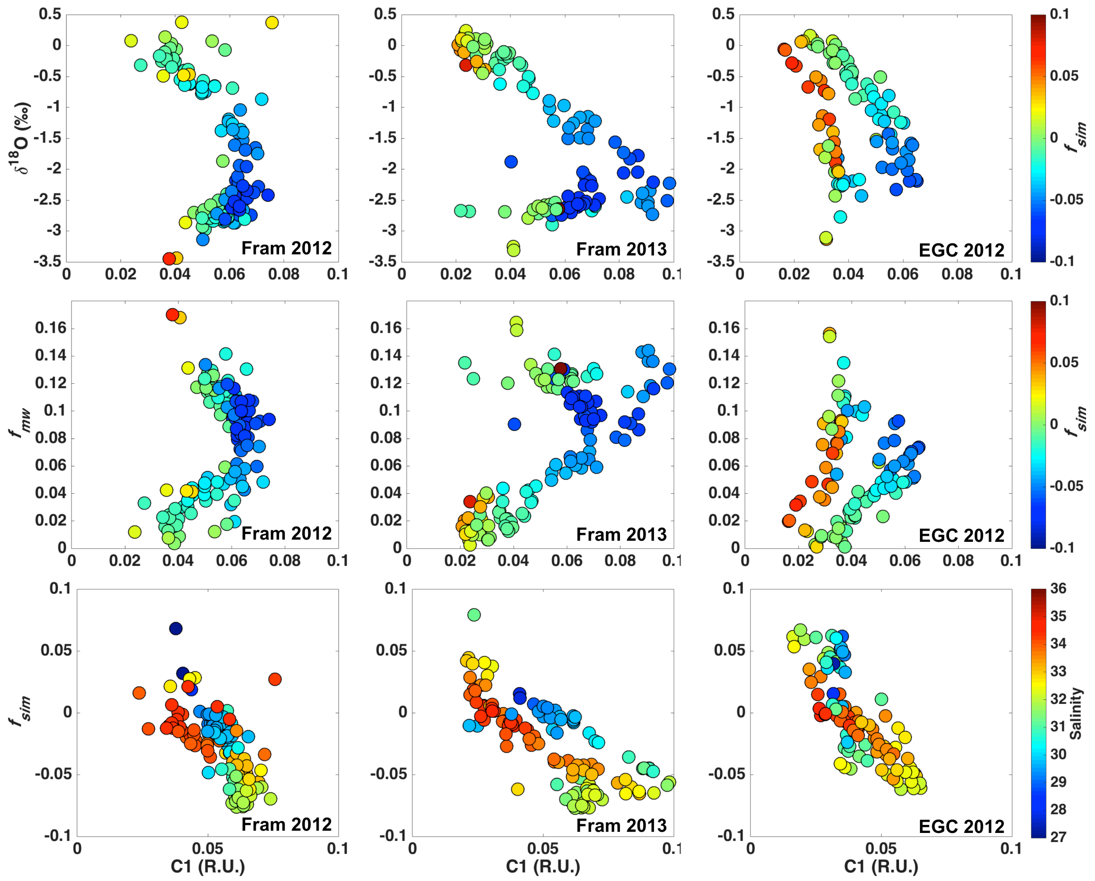

Figure S4. Vertical distribution of UV-fluorescent C3 (R.U.) along the transects. (a) Fram2012, (b) Fram2013 and (c) Davis2013. Note the differences in color bar ranges for the cruises. Produced with Ocean Data View [60].

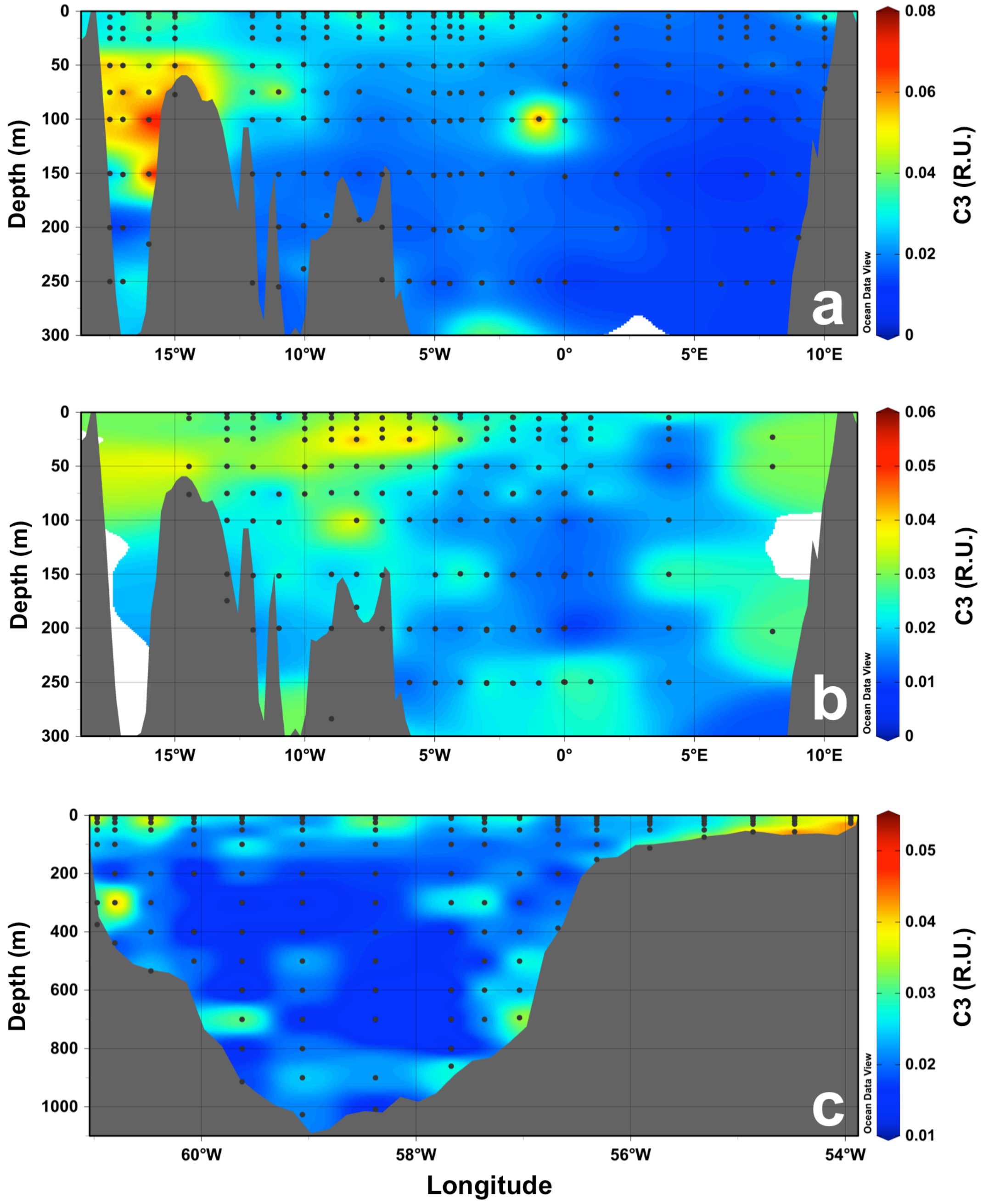

Supplement: Supplementary Information [file srep33978-s1.pdf]
